# Shoulder Dysfunction and Mobility Limitation in Aging

**DOI:** 10.20900/agmr20230008

**Published:** 2023-11-17

**Authors:** Derik L. Davis

**Affiliations:** Department of Diagnostic Radiology and Nuclear Medicine, University of Maryland School of Maryland, 22 S. Greene Street, Baltimore, MD 21042, USA

**Keywords:** aging, mobility, older adult, pain, rotator cuff, shoulder

## Abstract

Mobility limitation is common among older populations and is a major burden to public health. While lower extremity dysfunction is a known contributor, the influence of shoulder dysfunction on mobility is less well understood. Shoulder pain and rotator cuff tear are common causes of shoulder dysfunction, and both ailments are highly prevalent in older adults. This article discusses shoulder pain and rotator cuff tear as contributors to shoulder dysfunction and describes the association of shoulder dysfunction with mobility limitation in older adults.

## INTRODUCTION

Mobility limitation is a major burden on public health, affecting more than 1 in 3 older adults in the United States [1]. Mobility limitation is associated with loss of functional independence and is a marker for disability, falls, hospitalization, institutionalization, and mortality in older populations [2-8]. The association of poor lower extremity function with mobility limitation in older populations is well documented [9-11].

Investigators have called for development of more holistic research strategies to provide greater scientific understanding of reduced mobility in older populations [12]. For example, the United States National Institute on Aging convened a multi-disciplinary 2-day workshop in September 2021 to identify key gaps in knowledge and future innovative research aims for discovery of novel factors contributory to consequent mobility limitation in aging [12]. Identifying novel risk factors for decline in mobility performance and developing innovative interventions to prevent or to mitigate mobility limitation are logical next steps following the transformative LIFE-Pilot and LIFE Studies, which showed that novel risk factors and interventions for mobility limitation can be discovered and lead to improved long-term health outcomes for older populations [13].

The potential association of shoulder dysfunction with mobility limitation has been mostly overlooked and remains not well understood. Conceptually, the shoulder is the mobile link between the upper limb and trunk, facilitating arm swing during ambulation. Swinging of the arms during ambulation provides dynamic stability via coupled rhythmic interlimb coordination among the upper and lower extremities [14-16]. The shoulder also facilities common activities of daily living (ADLs) such as enabling load carriage while carrying a shopping bag or heavy object for example, necessary to accomplish ambulatory tasks and maintenance of functional independence [17-18]. Thus, when holistically considering human gait as dependent on upper extremity function, as well as lower extremity function, research investigating the contribution of shoulder dysfunction to mobility limitation in older populations is warranted.

## COMMON CAUSES OF SHOULDER DYSFUNCTION

Shoulder dysfunction is common among older adults and may accelerate functional decline [17,18]. Populations with shoulder dysfunction typically present with inferior scores for ADLs on shoulder-specific self-reported instruments and also decreased shoulder strength and range of motion (ROM) on physical examination [18-20]. Shoulder dysfunction is also detrimental to health-related quality of life in older populations [17,18,21]. Causes of shoulder dysfunction are multifactorial, with subjective shoulder pain a major contributor [22-25]. Objective structural abnormalities are also common, with rotator cuff (RC) tear as a leading cause [22,26].

### Shoulder Pain

Shoulder pain is highly prevalent in older populations. The 2011 National Health and Aging Study estimated a nearly 20% prevalence of pain at the shoulder among adults aged 65 years and older in the United States, only trailing the back (30%) and knee (25%) as the most common source of musculoskeletal pain [24]. Shoulder pain is associated with decreased ROM and strength at the shoulder [27]. The mere presence of shoulder pain and not the degree is the most important factor, as pain may not have an independent effect on physical function in older adults [28]. Shoulder pain is most commonly associated with RC disorders among clinical patients, although not all painful shoulders are caused by the rotator cuff [22,25,26].

The burden of shoulder dysfunction among older populations on public health is likely under-estimated. Nearly one-half of older clinical patients with chronic bouts of shoulder pain do not report their symptoms to their primary care providers [23,25]. Even high-functioning older adults, a population typically considered to be free of disability, may perceive pain-related physical limitation. McMahon et al. in a study of 132 Olympians aged 60 years and older at the 2005 Senior Olympics reported that participants with shoulder pain showed significantly inferior scores on self-reported surveys of shoulder function, relative to participants without shoulder pain [29].

However, pain is not required to harbor a functional deficit. Populations of older adults with asymptomatic RC tears display inferior ROM at the shoulder on physical examination compared to peers who lack RC tear [30,31].

### Rotator Cuff Tear

The rotator cuff is a myotendinous structure that provides dynamic stability at the shoulder. Working in synchronized concert with static stabilizers—the glenoid labrum, bone morphology, glenohumeral capsule-ligamentous complex, and negative intra-articular pressure, the rotator cuff facilitates stability of the glenohumeral joint through all phases of motion [32-35].

RC tear is the physical manifestation of an acquired anatomic defect in the tendon of the myotendinous unit which potentially compromises dynamic stability of the glenohumeral joint. RC tear is primarily a disease of aging [36,37]. Some investigators have postulated that RC disease is a primary determinant of health status among clinical patients [38,39]. The prevalence of RC tear is relatively low in populations <50 years and dramatically increases in older populations [37]. Most RC tears in older populations are atraumatic, occur during the performance of usual ADLs, and are predisposed by a weakened, aging tendon structure relative to younger populations. RC tear affects one in every four adults aged 60 years and older, with tear of the supraspinatus tendon the most often encountered [31,37,40]. Adults 80 years and older have a prevalence of RC tear as high as 50% [37].

Pain, limited ROM and weakness at the shoulder are common symptoms of RC tear, although not all individuals with RC experience symptoms [29]. Symptomatic RC tear is associated with decline in ADLs and physical function [18,41-43]. In a study of thoracohumeral shoulder motion using three-dimensional motion kinematics, Vidt et al. reported that older adults with symptomatic RC tear showed abnormal shoulder motion when asked to perform common ADL-related upper-extremity tasks relative to asymptomatic controls [41]. Theses investigators hypothesized that RC tear or shoulder pain avoidance manifested as muscle-force imbalance when attempting to complete ADL-related tasks [41].

### Rotator Cuff Imaging

**Figure 1.**
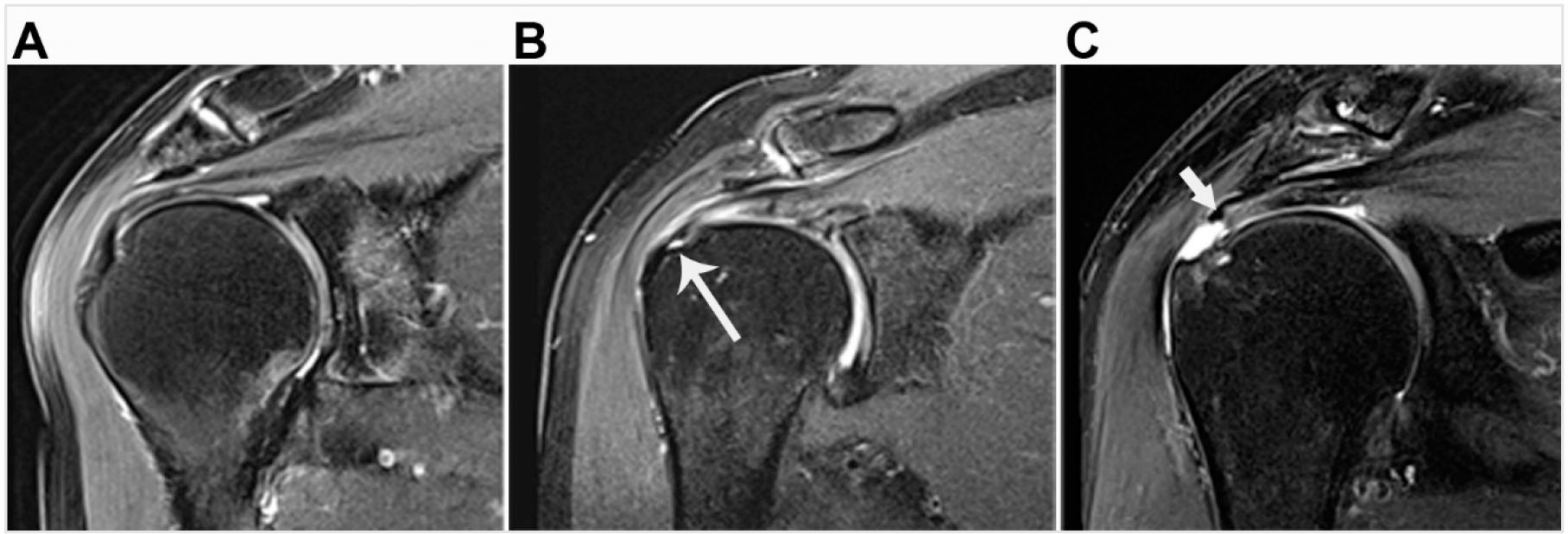
Examples of rotator cuff tendon classification on magnetic resonance imaging. (**A**) Supraspinatus tendon shows no sign of tear. (**B**) Partial-thickness tear of the supraspinatus tendon at its greater tuberosity humeral footprint (long arrow). (**C**) Full-thickness tear of the supraspinatus tendon with medial retraction of the tendon edge (short arrow) away from the greater tuberosity humeral footprint.

**Figure 2.**
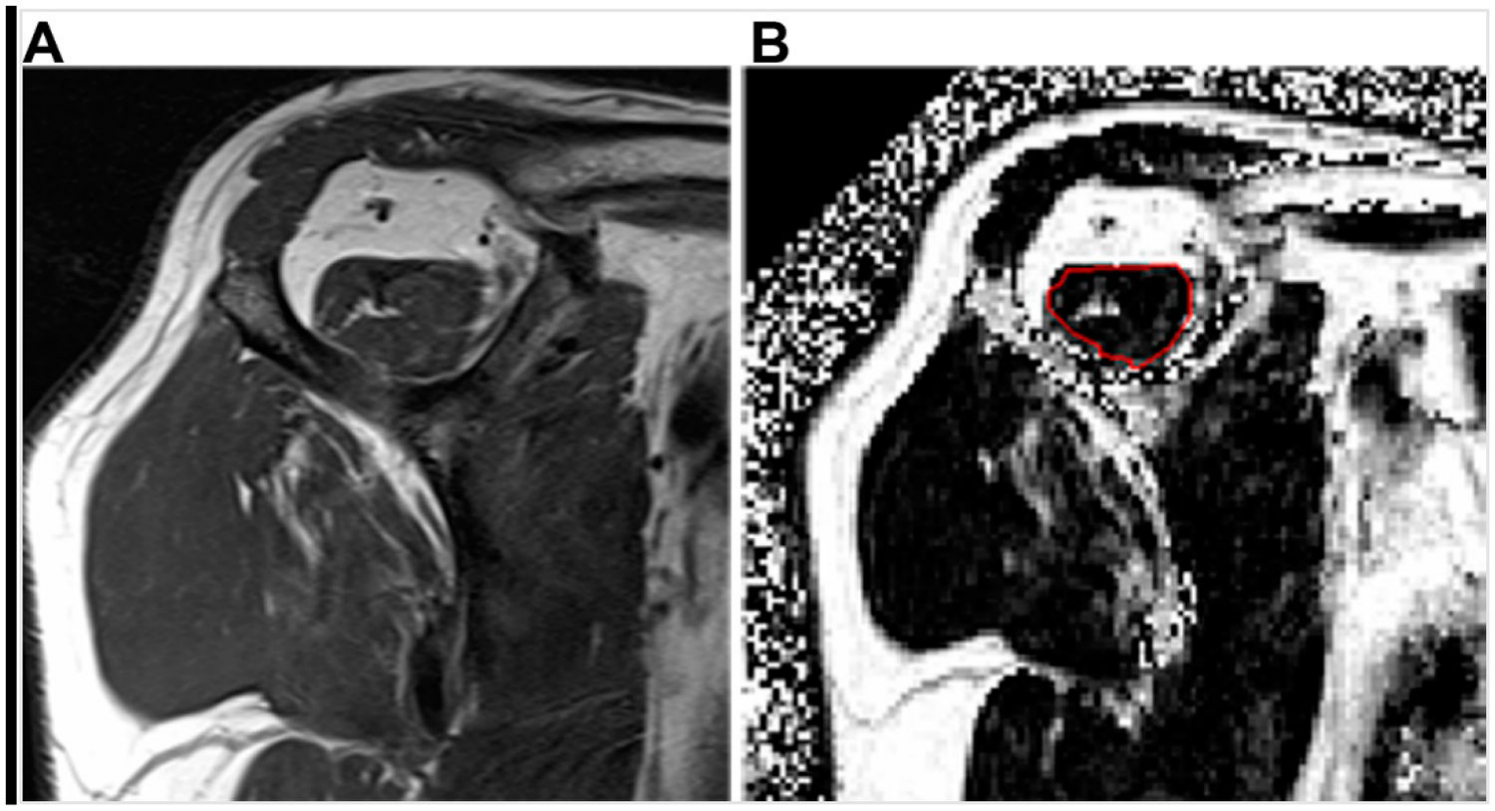
Examples of rotator cuff muscle fatty infiltration classification on magnetic resonance imaging (MRI). (**A**) Oblique sagittal T1-weighted MRI shows a supraspinatus muscle with streaks of internal fat, consistent with semi-quantitative Goutallier grade 1 fatty infiltration. (**B**) Quantitative oblique sagittal 6-point Dixon fat fraction map MRI following manual image segmentation calculates an amount of 10.5% supraspinatus muscle fatty infiltration within the region of interest (red circle).

Imaging is central for establishing the clinical diagnosis and prognosis for patients presenting with either traumatic acute-onset shoulder dysfunction or non-traumatic shoulder disorders which are not self-limited [22,44,45]. Magnetic resonance imaging (MRI) is the leading imaging modality in the United States to provide definitive imaging evaluation of patients with symptomatic shoulder dysfunction and is a valid and reliable method for diagnosing and characterizing the pattern of RC tear (Figure 1) [20,42,46-48]. MRI also provides concurrent prognostic information about the degree of intramuscular fatty infiltration associated with RC tear, a critical factor that influences orthopaedic surgeons’ clinical decision-making regarding patients’ eligibility to receive RC repair (RCR) surgery (Figure 2) [20,42,46-50]. RC tear is associated with structural changes in the corresponding muscle belly related to intramuscular proliferation of adipose cells and inflammation, fibrosis and apoptosis of myofibers leading to global muscle atrophy and dysfunction [51-53]. Animal and human models of RC tear show that intramuscular fatty infiltration is inversely related to muscular contractile force at the rotator cuff and does not improve after RCR surgery [20,51,54]. During initial steps of pre-operative evaluation, imaging metrics of RC muscle quality are critical, as advanced RC intramuscular fatty infiltration is a relative contraindication to RCR surgery and is associated with poor postsurgical outcomes [46,48-50].

## DIAGNOSIS OF SHOULDER DYSFUNCTION

The prevalence of shoulder dysfunction among older adults in the general population is likely under-estimated, as the number of older adults with symptoms of shoulder dysfunction who do not seek evaluation from healthcare providers is unknown. Given that nearly-one half of the population of older adults with non-acute shoulder dysfunction do not report shoulder complaints to their primary care provider [23,25], a reasonable hypothesis would be that a large number of older adults in the general population are not receiving treatment for their shoulder dysfunction from a healthcare provider.

Shoulder dysfunction’s effect on functional decline may be under-recognized by current screening paradigms for disability in older populations. Traditional self-reported screening tools used to screen for level of disability in older populations, such as the Katz ADLs and Lawton-Brody instrumental ADLs surveys, may exhibit ceiling effect and be insensitive to detect shoulder-specific disability in the population of community-dwelling older adults at large [17]. In contrast, upper extremity or shoulder-specific self-reported surveys may improve sensitivity for quantifying perceptions of performing ADLs in this population [17].

Practice standards vary among healthcare providers for diagnosis and treatment of older adults who present as clinical patients with chief complaint of shoulder symptoms. Older adults may initially seek care from a variety of healthcare professionals, including physical therapists, primary care providers, emergency medicine physicians, orthopaedic surgeons, sports medicine physician specialists, rheumatologists, and chiropractors. Physical therapists, for example, are often the first to encounter older adults with shoulder complaints through direct access, also known as patient self-referral without prior evaluation by another healthcare provider. Physical therapists most often establish a clinical diagnosis through history and physical examination alone [55-57]. Given that most physical therapists do not order imaging tests, treatment is most often rendered without explicit knowledge of the underlying anatomic pathology [19]. On the other hand, primary care providers often order shoulder imaging studies, but may lack the expertise to render an effective assessment or treatment plan relative to subspecialty physicians or rehabilitation specialists [55]. Given the lack of a universal standard of care amongst varying healthcare professions and also the lack of affiliation or coordination amongst providers, clinical treatment strategies for older adults with shoulder dysfunction are frequently a function of the type of healthcare professional initially encountered, which may not necessarily result in the most optimal approach.

## TREATMENT OF SHOULDER DYSFUNCTION

Treatment of shoulder dysfunction in clinical patients is dependent on presenting symptoms, which commonly include pain, weakness and decreased ROM [27,42,58,59]. Imaging tests help to stratify patients into two groups – those who may benefit from surgery and those for whom conservative measures are indicated, such as medication management with or without physical rehabilitation [19,55,58,60]. For example, older adults receiving physical therapy for shoulder dysfunction commonly present with inferior scores on self-reported surveys of shoulder function relative to control populations [19]. Goals of physical therapy may include decreasing shoulder pain and improving strength and ROM over time, often measured by shoulder-specific self-reported instruments of shoulder function [17-19].

Best treatment practices for RC tear among older persons is controversial, and non-operative management with physical therapy remains in a state of equipoise with RCR surgery [58,60-62]. The general goals of operative intervention for patients considered appropriate candidates for RCR surgery typically include reduction of pain and improvement in strength and ROM [20,42]. Orthopaedic surgeons consider older adults as potential candidates for RCR surgery on a case-by-case basis according to their own preference and a plethora of patient-specific factors, since there is no general consensus about best clinical practice for this age group [20,60,61,63]. Historically, orthopaedic surgeons were less inclined to offer RCR surgery to older populations relative to young or middle-aged populations [43,60,64]. Willingness among orthopaedic surgeons to offer RCR surgery to older persons is evolving in current clinical practice, and there has been a general trend of increased rates of operative intervention relative to non-operative management over the past few decades. There is a growing sentiment that older adults with symptomatic RC tear benefit from significant functional and clinical improvement following RCR surgery, similar to young adult and middle-aged populations [60,64]. In a study of Medicare recipients from 2005 to 2012, the percentage of patients with RC tear receiving RCR surgery rose from 33.8% to 40.4% while the percentage treated only by physical therapy fell from 30.0% to 13.2% [61]. The group of older adults, aged 65 to 74 years, demonstrate the highest annual rate of RCR surgery utilization among all age groups at 28.3 per 10,000 persons [65].

The annual cost of RCR surgery has been estimated to be as high as $12 billion dollars for the nearly 300,000 performed each year in the Unites States [66]. The burden of financial costs related to RC tear on the healthcare system are likely to increase further over time as older populations continue to grow over the coming decades and will be compounded by the fact that 4% of patients who receive RCR surgery will require operative revision within 1 year [27,67].

## RELEVANCE OF SHOULDER DYSFUNCTION FOR MOBILITY

Early detection of mobility limitation is an important factor to enable prevention or mitigation of subsequent mobility-related disability among community-dwelling older adults. Valid and reliable screening methods toward this goal improve healthcare providers’ ability to stratify this population by mobility performance. The Short Physical Performance Battery (SPPB), for example, is a well-known tool used in research and clinical practice to quantify lower extremity performance. The SPPB is a feasible performance-based categorical screening tool to quantify lower extremity function, on a scale from 0 (worst performance) to 12 (best performance). The SPPB score is the sum of 3 components, each scored on a scale of 0 (worst performance) to 4 (best performance) which rate static balance, chair rise, and gait speed performance, respectively [2,9]. A score < 10 is considered to represent mobility limitation [68]. The SPPB has been validated to predict development of subsequent mobility-related disability and hospitalization among populations of community-dwelling older adults who are non-disabled at baseline [2,10,11]. The SPPB also is strongly predictive of older adults’ ability to walk 400 meters [11].

Performance-based measures, such as the expanded Short Physical Performance Battery (eSPPB), were developed to raise the measurement ceiling of SPPB to evaluate a broader range of lower extremity performance on a continuous scale [69]. Researchers also have used individual subcomponents of SPPB as stand-alone metrics of lower extremity performance to predict outcomes in older populations. For example, in a pooled analysis of 34,485 community-dwelling older adults from 9 cohort studies, Studenski et al. reported that gait speed was associated with survival, showing that increased increments in gait speed were associated with increased survival [8].

The United States Center for Disease Control and Prevention promotes additional common feasible mobility performance screening instruments for older populations as part of its Stopping Elderly Accidents, Deaths & Injuries program. Examples include the 30-second chair stand test [70], a test of lower extremity strength and endurance that reduces floor effect relative to the 5 chair stand test of the SPPB, and the Timed Up and Go test [71,72].

**Figure 3.**
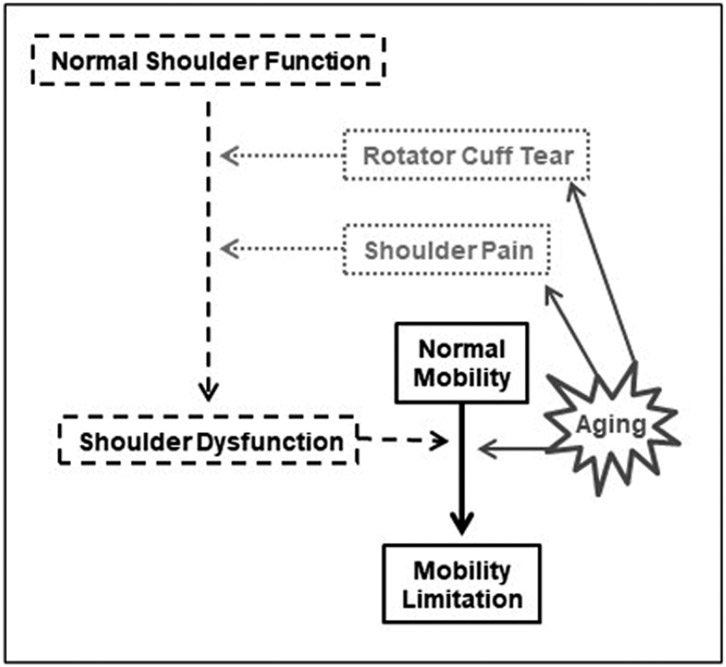
Conceptual model linking aging, mobility, shoulder dysfunction, rotator cuff tear, and shoulder pain.

Quantifying the contribution of shoulder dysfunction towards development of mobility limitation in community-dwelling older populations is a relatively novel concept, in line with the need to develop newer innovative, holistic strategies to prevent or to mitigate mobility limitation (Figure 3). Investigators have begun to test for association between shoulder measures and lower extremity function, since gaps in knowledge still currently exist.

In a cross-sectional study of 130 community-dwelling older adults, James et al. assessed the association of shoulder coordination with SPPB score by testing rhythmic interlimb coordination between shoulders using non-ambulatory function testing via auditory metronome tones in a supine position via three-dimensional motion capture kinematics. These investigators reported that study participants in the poorest quartile of shoulder coordination had a 6.7 times adjusted odds (*p* = 0.001) to manifest a SPPB score <10, relative to participants in the best quartile of shoulder coordination [73]. In a subsequent longitudinal study James et al. reported that impaired non-ambulatory shoulder coordination via auditory metronome tone testing at baseline was a significant risk factor for development of mobility limitation defined as an SPPB score <10 within the following 12 months, in a population of older adults who reported no musculoskeletal abnormality at the shoulders and had a SPPB score ≥10 at baseline [74].

Musculoskeletal pain and/or biomechanical structural abnormality local to the shoulder(s) also may abnormally influence arm swing and coordination of the extremities during ambulation, although there is a current knowledge gap in this area. Dysfunction local to the shoulder secondary to pain or structural abnormality, such as RC tear, has a known consequence of limited ROM or loss of strength [18,20,27,41,42]. In addition, shoulder glenohumeral joint loading is a normal part of ADLs among community-dwelling older adults [41], and shoulder dysfunction produced by carrying items also has the potential to impact mobility when performing routine tasks at home or in the community.

The link between shoulder ROM and mobility limitation in older populations is nascent in description. In a cross-sectional analysis of 613 participants 60 years or older in the Baltimore Longitudinal Study of Aging, Davis et al. examined the association of restricted shoulder elevation and external rotation ROM with measures of lower extremity performance and walking endurance capacity. These investigators found that participants who failed to demonstrate full ROM at both shoulders on physical examination had as high as a 4.5 times adjusted odds (*p* = 0.020) to perform poorly on the fast-paced 400-meter walk test and also as high as a 3.2 times adjusted odds (*p* = 0.004) to perform poorly on the eSPPB, relative to participants with full ROM at both shoulders [75].

Additional research is needed to further examine the strength of association between shoulder dysfunction and mobility performance measures relevant to the daily lives of community-dwelling older adults. One current study funded by the National Institute on Aging “Shoulder Pain, Rotator Cuff Tear, Coordination, and Mobility in Aging” is underway which seeks to establish preliminary data on the association of shoulder coordination while overground walking with mobility performance measures, and also the association between of shoulder coordination while walking overground with RC tear or shoulder pain, in a population of community-dwelling older adults [76].

## CONCLUSIONS

In conclusion, shoulder dysfunction and mobility limitation are common conditions that contribute to functional decline in older populations. The idea that lower extremity dysfunction does not fully explain all mechanisms of mobility limitation is gaining acceptance in the scientific community. Holistic research strategies that investigate the influence of shoulder dysfunction on mobility offer the potential for development of future novel interventions that improve prevention or mitigation of mobility limitation in older adults.

## Data Availability

No data were generated from this study.

## ABBREVIATIONS

ADLs: Activities of Daily Living
eSPPB: Expanded Short Physical Performance Battery
MRI: Magnetic Resonance Imaging
RC: Rotator Cuff
RCR: Rotator Cuff Repair
ROM: Range of Motion
SPPB: Short Physical Performance Battery
